# A novel splice site mutation in the *UBE2A* gene leads to aberrant mRNA splicing in a Chinese patient with X‐linked intellectual disability type Nascimento

**DOI:** 10.1002/mgg3.976

**Published:** 2019-09-30

**Authors:** Dingyuan Ma, Jianxin Tan, Jing Zhou, Jingjing Zhang, Jian Cheng, Chunyu Luo, Gang Liu, Yuguo Wang, Zhengfeng Xu

**Affiliations:** ^1^ State Key Laboratory of Reproductive Medicine Department of Prenatal Diagnosis Women's Hospital of Nanjing Medical University Nanjing Maternity and Child Health Care Hospital Nanjing People's Republic of China

**Keywords:** splicing mutation, *UBE2A*, whole exome sequencing, X‐linked intellectual disability

## Abstract

**Background:**

X‐linked intellectual disability type Nascimento (XIDTN), caused by mutations in ubiquitin‐conjugating enzyme E2A (*UBE2A*) gene, is characterized by moderate to severe intellectual disability, impaired speech, urogenital anomalies, skin abnormalities, and dysmorphic facial features.

**Methods:**

Whole‐exome sequence was carried out in the patients, and the variant of disease‐associated gene in the patient and his parents was confirmed by Sanger sequencing. RNA transcript analysis by reverse transcription (RT)‐PCR was performed to assess the potential effects of the splice site mutation.

**Results:**

A novel splicing mutation (c.331‐2A>G) in *UBE2A* gene, inherited from his mother, was identified in a Chinese boy with intellectual disability and impaired speech. Furthermore, brain magnetic resonance imaging showed multiple patchy hyperintensity in bilateral centrum ovale. RT‐PCR demonstrated that this variant generated a novel transcript with a deletion of 29 nucleotides in exon 6 (r.331_359del), resulting in a frameshift mutation (p.L112SfsX17).

**Conclusion:**

Ultimately, he was diagnosed with XIDTN by genetic analysis. To the best of our knowledge, this is the first case report of this syndrome in China with a confirmed molecular diagnosis. Our case not only expands the mutation spectrum of *UBE2A*, but also provides additional insights into the genetic and phenotypic heterogeneity of XIDTN as well as phenotype–genotype correlations in this disease.

## 1 INTRODUCTION

X‐linked intellectual disability type Nascimento (XIDTN) (MIM 300860), also known as ubiquitin‐conjugating enzyme E2A (*UBE2A*, OMIM: 312180) deficiency syndrome, is a rare, syndromic intellectual disability characterized by moderate to severe intellectual disability, impaired speech, urogenital anomalies, skin abnormalities, and dysmorphic facial features (Czeschik et al., 2013; Thunstrom, Sodermark, Ivarsson, Samuelsson, & Stefanova, 2015). This disorder, first delineated by Nascimento, Otto, de Brouwer, & Vianna‐Morgante (2006), is caused by intragenic mutations or deletions encompassing the *UBE2A* gene. *UBE2A*, located on Xq24, encodes a ubiquitin‐conjugating enzyme, which is essential for the ubiquitin proteasome pathway of protein degradation and contributes to DNA repair, fertility, and memory formation (Bruinsma et al., 2016; Nandi, Tahiliani, Kumar, & Chandu, 2006). *UBE2A* deficiency syndrome occurs exclusively in males, and the mothers of the patients show skewed X‐inactivation and are clinically unaffected. To date, at least 19 patients with intragenic *UBE2A* mutations and 12 patients carrying microdeletions encompassing *UBE2A* have been described (Giugliano et al., 2018; Tsurusaki et al., 2017). So far, there has been no report of *UBE2A* deficiency syndrome in China.

Occurring prior to mRNA translation, RNA splicing is an essential post‐transcriptional process to remove introns from pre‐mRNA and connect adjacent exons together, in which the consensus intronic dinucleotide splice donor, GT and the splice acceptor, AG are used to precisely identify exon–intron boundaries (Havens, Duelli, & Hastings, 2013). It is relatively straightforward to interpret the mutations in the canonical dinucleotide splice donor/acceptor sequences because they have been presumed to be invariably deleterious (Caminsky, Mucaki, & Rogan, 2014). In general, the modifications in the canonical splice sites may lead to exon skipping, cryptic splice site activation or intron retention and ultimately exert detrimental effects on protein functions (Kapustin et al., 2011). To confirm these potential splicing effects, transcription products need to be analyzed experimentally.

Herein, we present a Chinese boy with X‐linked intellectual disability carrying a novel acceptor splice site mutation in intron 5 (c.331‐2A>G) of the *UBE2A* gene (reference sequence: NM_003336.3). Furthermore, RNA transcript analysis by reverse transcription (RT)‐PCR demonstrated that this variant completely abolishes the natural acceptor sequence by creating a novel 3’ splice acceptor site and generates a novel transcript with a deletion of 29 nucleotides in exon 6 (r.331_359del) and presumably protein truncation.

## 2 MATERIALS AND METHODS

### 2.1 Ethical compliance

The written informed consent was obtained from his parents, and all procedures were reviewed and approved by the Ethics Committee of Nanjing Maternity and Child Health Care Hospital (Nanjing, Jiangsu, China).

### 2.2 Subjects

The proband is a 5‐year‐old boy of Chinese origin who was admitted because of limited walking, protruding tongue movements, speech impairment, and intellectual disability. Peripheral blood samples were collected from the proband and his parents.

### 2.3 Genomic DNA analysis

Genomic DNA was isolated from whole blood by using an Automated Nucleic Acid Extractor (RBCBioscience). Whole‐exome capture was carried out with an Agilent SureSelect Human All Exon v6 kit according to the manufacture's protocol. The enriched exome libraries were sequenced on an Illumina Novaseq 6000 platform (Illumina). Sequence alignment against the reference human genome (GRCh37) was performed using Speedseq (Chiang et al., 2015). Single‐nucleotide variants (SNVs) and small indels (<10 bp) were identified using GATK HaplotypeCaller (McKenna et al., 2010), and filtered according to the Broad Institute's best‐practice guidelines (version 3). Filter‐passed variants were annotated with the ANNOVAR software (Wang, Li, & Hakonarson, 2010). Allele frequency was assessed using the dbSNP147, 1000 Genomes (the 1000 Genomes Project), ESP6500 (NHLBI Exome Sequencing Project), ExAC (the Exome Aggregation Consortium) and gnomAD (the Genome Aggregation Database). Reported pathogenicity was assigned using Human Gene Mutation Database and ClinVar. The effects of SNVs were predicted by SIFT, Polyphen‐2, MutationTaster, MutationAssessor, FATHMM, and RadialSVM programs. The potential splicing effects were checked by using splice‐site prediction software, including MutationTaster (http://www.mutationtaster.org), NNSplice (http://www.fruitfly.org/about/index.html), and scSNV algorithm (Jian, Boerwinkle, & Liu, 2014). Sequence variants were classified according to the American College of Medical Genetics and Genomics‐Association for Molecular Pathology (ACMG‐AMP) guidelines (Richards et al., 2015). PCR was performed to amplify *UBE2A* gene intron 5 that includes the variant identified by whole exome sequencing. PCR products were purified and subjected to direct DNA sequencing on an automated DNA Analyzer (3730 Genetic Analyzer; Thermo Fisher Scientific).

### 2.4 RNA transcript analysis by RT‐PCR

Total RNA was extracted from peripheral leukocytes by using TRIzol^™^ LS Reagent (Invitrogen; Thermo Fisher Scientific) according to the manufacture's recommended instructions. The concentrations and quality of total RNA were assessed by measuring the absorbance at 260 and 280 nm. RT was carried out to synthesize cDNA using a PrimeScript™ RT reagent Kit with gDNA Eraser (TAKARA). cDNA was amplified by using specific primers that cross *UBE2A* gene intron 5, and the PCR products were separated on a 2% agarose gel containing 0.5 mg/ml GeneFinder^™^ (Zeesan Biotech). The sequences of the primers will be provided upon request.

## 3 RESULTS

**Figure 1:**
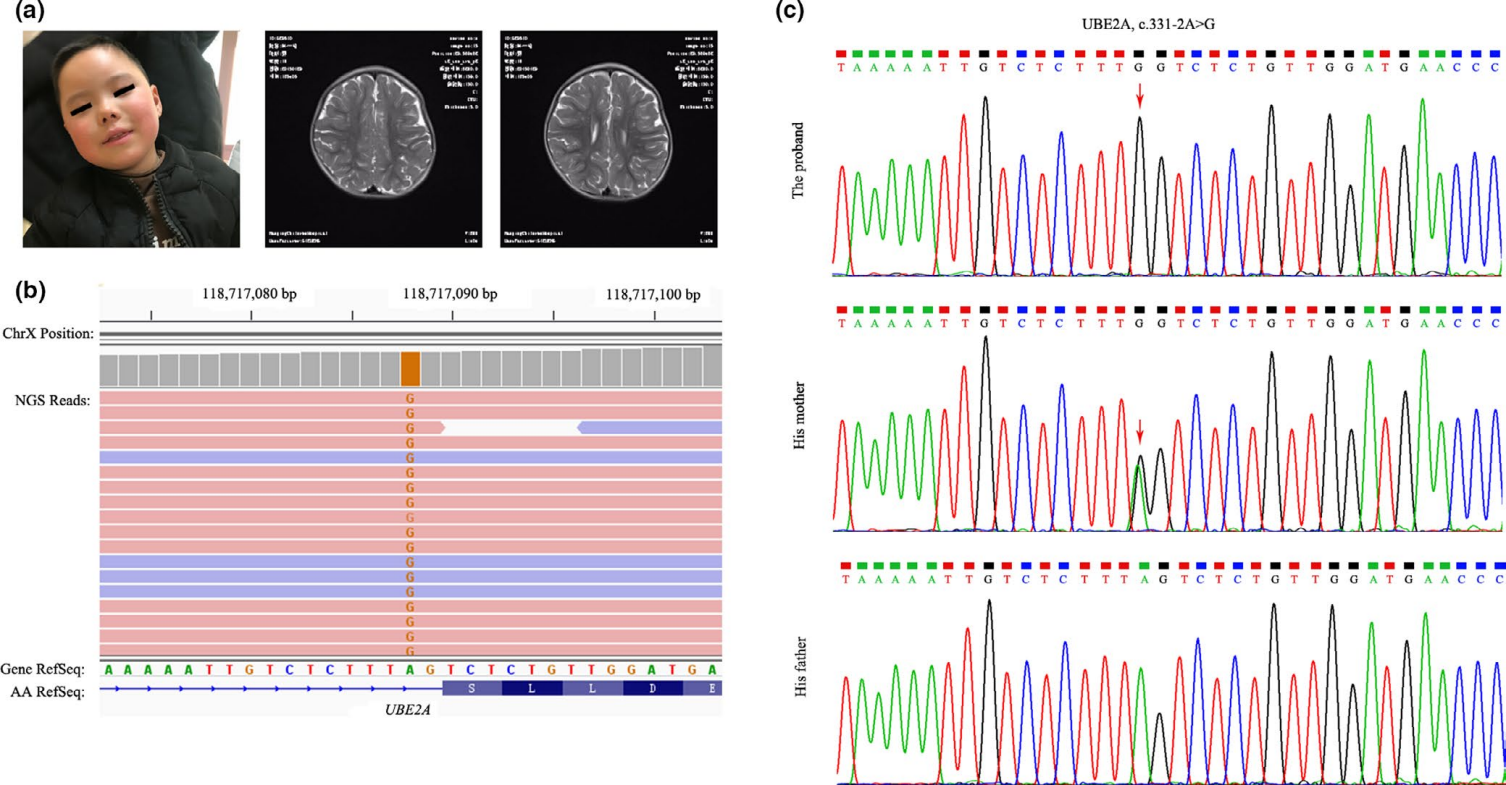
(a) Photograph of the proband (Left panel). Brain magnetic resonance images of the proband (Middle and right panel). (b) The mutation in *UBE2A* identified by NGS. (c) Confirmation of the mutation by Sanger sequencing. Reference sequence for *UBE2A* gene: NM_003336.3

The proband, a 5‐year‐old boy, was born to healthy nonconsanguineous Chinese parents after an uncomplicated term pregnancy. He was referred to the hospital because of severe developmental delay and intellectual disability. At 5 years of age, he could not walk unsupported or speak. His craniofacial findings included almond‐shaped eyes and protruding tongue movements (Figure 1a, left panel). However, he did not have seizures, and his general health was reasonably good without intercurrent hospitalizations. Brain magnetic resonance imaging showed multiple patchy hyperintensity in the bilateral centrum ovale (Figure 1a, middle and right panel).

Genetic analysis for intellectual disability was performed for the proband after consultation with a clinical geneticist. By using whole‐exome sequencing, a total of 78.3 million clean reads was obtained, with average sequencing depth of 98× and 96.5% of whole exome target region covered at ≥30×. In total, we detected 55,376 variants (SNVs and Indels). First, we screened 7989 rare variants with a minor allele frequency of ≤0.005 in 1000 Genomes, ExAC and gnomAD. After filtering out variants with no predicted or known functional consequence, we retained 842 variants. Given the neurobiological phenotypes of the affected boy, we filtered data so that we would retain the genes associated with intellectual disability. The gene list contained 881 genes with ‘Green’ status (meaning high diagnostic‐grade level of evidence) within the PanelApp Intellectual Disability panel (Version 2.4) curated by Genomics England (https://panelapp.genomicsengland.co.uk/panels/285/). Our filtering strategy finally resulted in 16 candidate variants, including 14 missense mutations, 1 in‐frame insertion, and 1 splicing mutation. We visually inspected these variants in the 16 known genes by using the Integrative Genomics Viewer software (Broad Institute; https://www.broadinstitute.org/igv). For classifying these variants, the allele frequencies from public databases and computational prediction results from in silico tools are shown in Table S1. The *UBE2A* c.331‐2A>G variant is located in the canonical splice site where loss of gene function is known mechanism of the disease (ACMG variant evidence PVS1). It has not been reported previously and is absent in 1000 Genomes, ExAC and gnomAD databases (ACMG variant evidence PM2). It was predicted as deleterious by in silico algorithms (MutationTaster, NNSplice and scSNV) (ACMG variant evidence PP3). Therefore, *UBE2A* c.331‐2A>G splicing was classified as pathogenic according to the ACMG guidelines. Among these variants, one variant was classified as likely benign, and the other 14 variants were of uncertain significance.

Whole‐exome sequencing in the proband identified a novel hemizygous mutation in *UBE2A* gene (c.331‐2A>G), and Sanger sequence confirmed that this mutation was inherited from his mother, who carried a heterozygous substitution for this variant (Figure 1b and c). This mutation was intronic and located at the intron–exon boundary, a potential splicing acceptor site.

**Figure 2:**
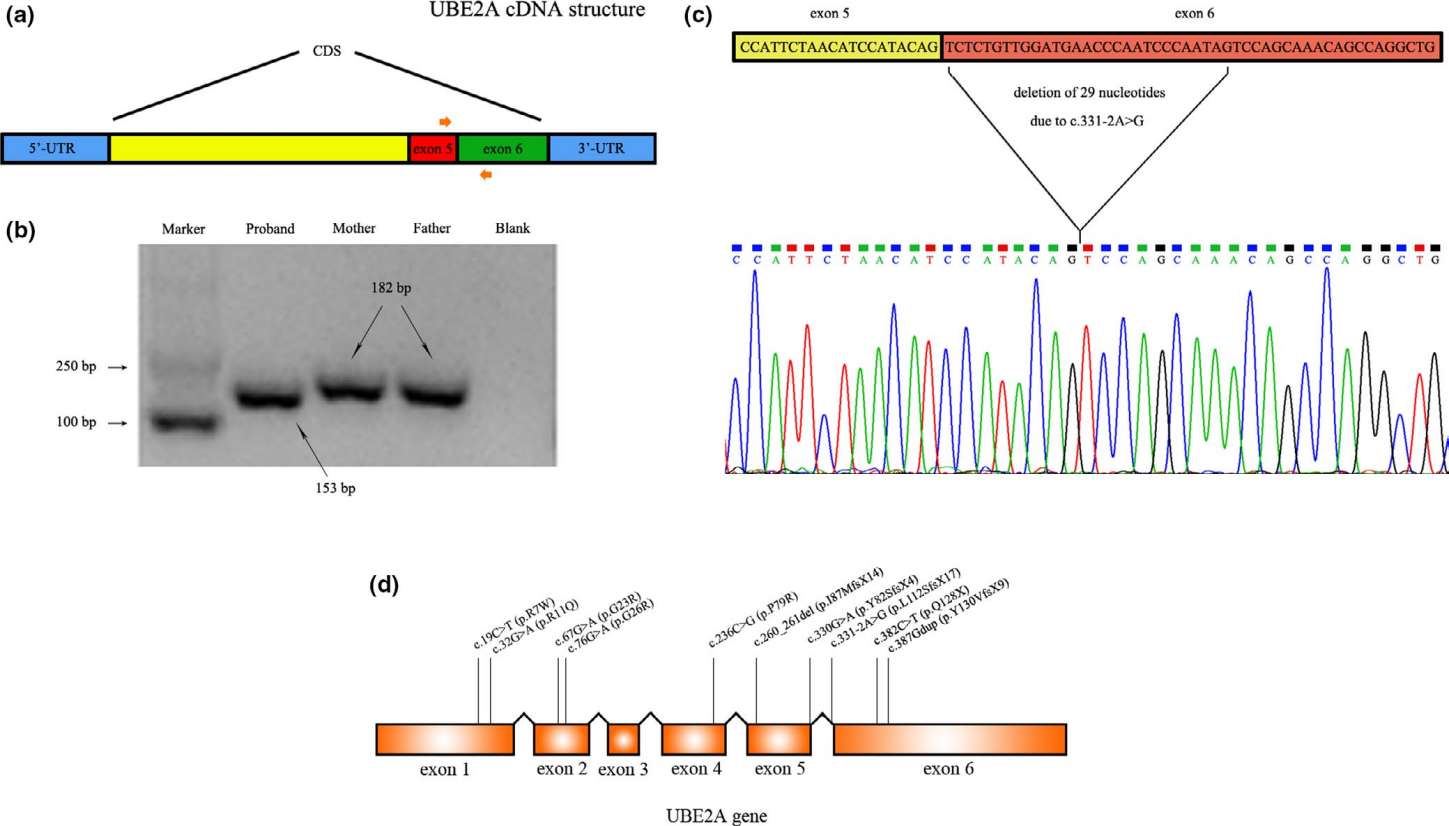
(a) Gene structure of *UBE2A* cDNA. (b) Gel image showing the PCR products of *UBE2A* cDNA fragments. (c) The deletion of 29 nucleotides in *UBE2A* mRNA was confirmed by Sanger sequencing. (d) Gene structure of the *UBE2A* gene and mutations described here and in previous reports. Reference sequence for *UBE2A* gene: NM_003336.3

Next, total RNA was extracted from the peripheral leukocytes of the proband and his parents, and PCR assays were performed to investigate the effects of this mutation on the splicing of *UBE2A* exon 6. As a result, the PCR product of the proband was smaller than that of his parents, and no normal sized band was observed (Figure 2a and b). The sequence analysis of the smaller product confirmed the presence of the deletion of 29 nucleotides at the beginning of exon 6 (r.331_359del), which was probably the consequence of a pseudo acceptor splice site in exon 6 (Figure 2c). The deletion of 29 nucleotides is predicted to cause a shift in the reading frame in *UBE2A* and ultimately introduces a premature stop codon at amino acid 128 in the 152‐amino‐acid full‐length protein.

So far, 10 intragenic *UBE2A* mutations have been reported among 20 patients from 10 families (including this study) diagnosed as XIDTN. The clinical features and genetic mutations of these 10 families from various origins are summarized in Table 1. All patients were male, and the mean age of the last diagnosis was 21.6 ± 14.1 (range 5–46 years). All patients showed moderate or severe intellectual disability and speech impairment. Other symptoms include synophrys (15/20), small penis (11/18), seizures (8/19), low nasal bridge (9/20), ocular hypertelorism (4/15), and low posterior hairline (6/12). In addition, white matter abnormalities were observed in 5 out of 10 patients. Of the 10 intragenic *UBE2A* mutations, five were missense, four frameshift, and one nonsense (Figure 2d).

**Table 1:** Clinical manifestations and molecular details for all reported patients with *UBE2A* mutations

References	Nascimento et al. (2006)		Czeschik et al. (2013)				Tsurusaki et al. (2017)	Budny et al. (2010)					Haddad et al. (2013)					Giugliano et al. (2018)	
Patients	1	2	3	4	5	6	7	8	9	10	11	12	13	14	15	16	17	18	19
Gender	M	M	M	M	M	M	M	M	M	M	M	M	M	M	M	M	M	M	M
Ages (years)	46	19	5	7	15	38	8	7	21	43	29	43	15	33	13	9	13	36	26
Ethnicity	Brazilian		Caucasian				Japanese	Caucasian					Caucasian					Caucasian	
Mutation	c.382C>T (p.Q128X)		c.387Gdup (p.Y130VfsX9)		c.236C>G (p.P79R)		c.76G>A (p.G26R)	c.67G>A (p.G23R)				c.32G>A (p.R11Q)	c.260_261del (p.I87MfsX14)		c.19C>T (p.R7W)			c.330G>A (p.Y82SfsX4)	
Speech impairment	+	+	+	+	+	+	+	+	+	+	+	+	+	+	+	+	+	+	+
Intellectual disability	+	+	+	+	+	+	+	+	+	+	+	+	+	+	+	+	+	+	+
Seizures	+	+	+	+	−	+	NA	+	+	−	+	−	−	−	−	−	−	−	−
White matter abnormalities	NA	+	+	−	−	−	+	NA	NA	NA	NA	NA	−	NA	NA	−	+	NA	NA
Synophrys	+	+	+	+	+	+	−	+	+	+	+	+	+	+	−	−	−	+	+
Ocular hypertelorism	−	+	−	+	−	−	−	−	−	−	−	−	NA	NA	NA	NA	NA	+	+
Low nasal bridge	−	+	+	+	−	+	+	−	+	−	−	−	+	−	−	+	−	+	+
Low posterior hairline	+	+	+	NA	−	+	NA	−	+	+	−	+	NA	NA	NA	NA	NA	−	−
Small penis	+	+	+	+	−	−	NA	−	+	+	+	+	−	−	−	+	+	+	NA

Note

Reference sequence for *UBE2A* gene: NM_003336.3

Abbreviations: M, male; +, present; −, absent; NA, not available.

## 4 DISCUSSION

In this study, we used exome sequencing to identify a novel *UBE2A* splice mutation, c.331‐2A>G, in a Chinese patient with intellectual disability. This mutation resulted in no detectable normally spliced *UBE2A* at the mRNA level and was predicted to produce a smaller aberrant transcript and presumably protein truncation. To the best of our knowledge, this is the first case report of this syndrome in China with a confirmed molecular diagnosis and a mild phenotype.

The *UBE2A* gene (also known as HHR6A or RAD6A) encodes a ubiquitin‐conjugating enzyme, which interacts with the ligating enzyme E3 to ubiquitinate mitochondrial proteins and facilitate proper elimination of dysfunctional mitochondria (Nandi et al., 2006). *UBE2A* gene is a highly evolutionarily conserved among mammals, and any minor alterations that impair functional domain of *UBE2A* may contribute to permanent disturbance in ubiquitination process and therefore be responsible for syndromic phenotype (Bruinsma et al., 2016). Moderate to severe intellectual disability and speech impairment have been reported in all patients with intragenic *UBE2A* mutations, including our patient. Intellectual disability, at least in part, results from defects in synaptic transmission. Haddad and colleagues have documented that loss of dRad6 function leads to reduced synaptic transmission because of mitochondrial failure (Haddad et al., 2013). Furthermore, they also demonstrated that RAD6A acts as a regulator of Parkin‐dependent mitophagy and plays a crucial role in maintaining neuronal function (Haddad et al., 2013). Therefore, mitochondrial dysfunction resulting from *UBE2A* mutations may lead to defects in synaptic transmission and intellectual disability in *UBE2A* deficiency syndrome. However, the underlying mechanisms by which *UBE2A* mutations affect neuronal function remain to be elucidated. It has been reported that the mothers of the XIDTN patients show skewed X‐inactivation (Czeschik et al., 2013; Tzschach et al., 2015). In the present study, direct RT‐PCR amplification of mRNA from the peripheral leukocytes of the mother yielded a product from only the wild‐type allele, suggesting that the mother has completely skewed X‐inactivation toward the mutated allele.

In this study, we are unable to obtain the brain tissues from the patient. Alternatively, we analyzed the splicing of *UBE2A* in blood leucocytes from the proband. Previous reports have documented that *UBE2A* is constitutively expressed in human brain tissue and blood leucocytes, and acts as a central effector in the ubiquitin proteasome pathway (Rey et al., 2018; Zhao, Alexandrov, Jaber, & Lukiw, 2016). In addition, all the isoforms of *UBE2A* contain the exon 6, which is disrupted by the splicing mutation (c.331‐2A>G) in mRNA splicing process. Thus, it is plausible to propose that aberrant splicing of *UBE2A* also occurs in brain tissue and contributes to the clinical phenotype of our patient.

To date, there have been 10 intragenic *UBE2A* mutations among 20 patients from 10 families (including this study) diagnosed as XIDTN (Giugliano et al., 2018; Tsurusaki et al., 2017). Unlike previous reports, our milder patient with *UBE2A* splice mutation presented with intellectual disability, speech impairment, and white matter abnormalities, without skin anomalies, urogenital abnormalities, seizures, and dysmorphic facial features. In our patient, the c.331‐2A>G mutation was precited to cause a truncated protein that deletes the N‐terminal of the ubiquitin‐conjugating enzyme catalytic (UBCc) domain, leading to reduced protein and/or activity levels. Bruinsma et al. (2016) have reported that Ube2a knockout mice have impaired hippocampal learning, which is similar to intellectual disability in our and previously described patients with *UBE2A* mutations. Furthermore, over a half of previous patients showed motor delay and seizures, but these phenotypes were not observed in Ube2a knockout mice (Bruinsma et al., 2016). *UBE2A* deficiency has emerged as a clinically well‐recognizable syndrome, but remains still underdiagnosed because of the limited number of patients reported and the variations of the clinical spectrum among patients. Therefore, more patients are needed to establish phenotype–genotype correlations.

In conclusion, we identified a novel RNA‐splicing mutation (c.331‐2A>G) in *UBE2A* gene resulting in an aberrant transcript and presumably protein truncation in a Chinese patient with XIDTN. In our milder patient, *UBE2A* splice mutation leads to a truncated protein, in which the N‐terminal of the UBCc domain is deleted. The role of the UBCc domain in neuronal function needs to be explored. Thus, the detailed molecular and clinical characteristics not only expand the mutation spectrum of *UBE2A*, but also provides additional insights into the genetic and phenotypic heterogeneity of XIDTN as well as phenotype–genotype correlations in this disease.

## CONFLICT OF INTEREST

The authors declare that they have no competing interests.
